# Fetal sex and risk of pregnancy-associated malaria in *Plasmodium falciparum-*endemic regions: a meta-analysis

**DOI:** 10.1038/s41598-023-37431-3

**Published:** 2023-06-26

**Authors:** Holger W. Unger, Anastasia Jessica Hadiprodjo, Julie R. Gutman, Valerie Briand, Nadine Fievet, Innocent Valea, Halidou Tinto, Umberto D’Alessandro, Sarah H. Landis, Feiko Ter Kuile, Peter Ouma, Martina Oneko, Victor Mwapasa, Laurence Slutsker, Dianne J. Terlouw, Simon Kariuki, John Ayisi, Bernard Nahlen, Meghna Desai, Mwayi Madanitsa, Linda Kalilani-Phiri, Per Ashorn, Kenneth Maleta, Antoinette Tshefu-Kitoto, Ivo Mueller, Danielle Stanisic, Jordan Cates, Anna Maria Van Eijk, Maria Ome-Kaius, Elizabeth H. Aitken, Stephen J. Rogerson

**Affiliations:** 1 Department of Obstetrics and Gynaecology, Royal Darwin Hospital, Darwin, NT Australia; 2 Menzies School of Health Research, Charles Darwin University, Darwin, NT Australia; 3 Department of Clinical Sciences, Liverpool School of Tropical Medicine, Liverpool, UK; 4 Department of Medicine (RMH), Peter Doherty Institute for Infection and Immunity, The University of Melbourne, Melbourne, VIC Australia; 5 Malaria Branch, Division of Parasitic Diseases and Malaria, Center for Global Health, U.S. Centers for Disease Control and Prevention, Atlanta, GA USA; 6 Université de Paris, UMR261, IRD, Paris, France; 7 Epicentre MSF, Paris, France; 8 Unite de Recherche Clinique de Nanoro, Institut de Recherche en Sciences de La Santé-DRCO, Nanoro, Burkina Faso; 9 Departement de Recherche Clinique, Centre Muraz, Bobo-Dioulasso, Burkina Faso; 10 Medical Research Council Unit, The Gambia at the London School of Hygiene and Tropical Medicine, Fajara, Gambia; 11 London School of Hygiene and Tropical Medicine, London, UK; 12 BioMarin Pharmaceutical, London, UK; 13 Kenya Medical Research Institute (KEMRI)/Centre for Global Health Research, Kisumu, Kenya; 14 School of Public Health and Family Medicine, College of Medicine, University of Malawi, Blantyre, Malawi; 15 Malaria and Neglected Tropical Diseases, Center for Malaria Control and Elimination, PATH, Seattle, WA USA; 16 Malawi-Liverpool-Wellcome Trust Clinical Research Programme, Blantyre, Malawi; 17 President’s Malaria Initiative, Washington, DC USA; 18 Malawi University of Science and Technology, Thyolo, Malawi; 19 Faculty of Medicine and Health Technology, Center for Child, Adolescent and Maternal Health Research, Tampere University, Tampere, Finland; 20 Department for Pediatrics, Tampere University Hospital, Tampere, Finland; 21 School of Public Health, University of Kinshasa, Kinshasa, Democratic Republic of Congo; 22 Walter and Eliza Hall Institute, Parkville, VIC Australia; 23 Institute for Glycomics, Griffith University, Gold Coast, QLD Australia; 24 Department of Epidemiology, UNC-Chapel Hill, Chapel Hill, NC USA; 25 Papua New Guinea Institute of Medical Research, Goroka, Papua New Guinea; 26 Department of Infectious Diseases, Peter Doherty Institute for Infection and Immunity, The University of Melbourne, Melbourne, VIC Australia; 27 Department of Microbiology and Immunology, Peter Doherty Institute for Infection and Immunity, The University of Melbourne, Melbourne, VIC Australia

**Keywords:** Epidemiology, Parasitology

## Abstract

In areas of moderate to intense *Plasmodium falciparum* transmission, malaria in pregnancy remains a significant cause of low birth weight, stillbirth, and severe anaemia. Previously, fetal sex has been identified to modify the risks of maternal asthma, pre-eclampsia, and gestational diabetes. One study demonstrated increased risk of placental malaria in women carrying a female fetus. We investigated the association between fetal sex and malaria in pregnancy in 11 pregnancy studies conducted in sub-Saharan African countries and Papua New Guinea through meta-analysis using log binomial regression fitted to a random-effects model. Malaria infection during pregnancy and delivery was assessed using light microscopy, polymerase chain reaction, and histology. Five studies were observational studies and six were randomised controlled trials. Studies varied in terms of gravidity, gestational age at antenatal enrolment and bed net use. Presence of a female fetus was associated with malaria infection at enrolment by light microscopy (risk ratio 1.14 [95% confidence interval 1.04, 1.24]; P = 0.003; n = 11,729). Fetal sex did not associate with malaria infection when other time points or diagnostic methods were used. There is limited evidence that fetal sex influences the risk of malaria infection in pregnancy.

## Introduction

Malaria is an infectious disease caused by parasites of the genus *Plasmodium* and transmitted by infected female *Anopheles* mosquitoes^1^. In 2020, about 241 million people had malaria and 627,000 died. The same year, 11.6 million pregnant women were exposed to malaria in sub-Saharan Africa, and malaria infection resulted in 819,000 neonates with low birth weight (LBW, < 2500 g)^2^. Most malarial infections worldwide are attributed to *Plasmodium falciparum* and *Plasmodium vivax*, with *P. falciparum* accounting for most of the morbidity and mortality, especially in sub-Saharan Africa. In Africa, it is estimated that a quarter of cases of severe anaemia in pregnant women and a fifth of LBW and stillbirth cases can be linked to malaria^3–5^. Of note, *P. falciparum*-infected erythrocytes can sequester in the placental intervillous space, and placental malaria is associated with LBW and stillbirth^3^.

The risk of malaria in pregnancy is modulated by different factors. Transmission intensity drives the development of acquired immunity, and this leads to differences in disease risk and severity between areas of high and low transmission^3^. Furthermore, parity-specific immunity acquired through pregnancies seems to contribute to a lower risk of placental malaria and the poor birth outcomes associated with it ^3^. Young maternal age may be an independent risk factor, as studies have shown that younger women are more susceptible to placental malaria and its poor outcomes, compared to older women with the same gravidity^6^.

There have been several studies exploring if and how fetoplacental sex might impact maternal health^7,8^. Male fetal sex has been associated with pregnancy complications such as term pre-eclampsia and gestational diabetes in meta-analysis of studies including over 12.5 million women^8^. Mothers of male fetuses are at increased risk of pre-eclampsia, but male fetuses may be more likely to maintain their growth trajectory compared to female fetuses. The mechanism could involve redirecting maternal blood flow to the placenta through vasoconstriction of maternal microvasculature, which is enhanced in pre-eclamptic compared normotensive women with a male fetus^9,10^. In contrast, there was no difference in the microvasculature of normotensive and pre-eclamptic women carrying female fetuses^9^. A study of asthma in pregnancy also demonstrated reduced growth in female compared to male fetuses. In the same study, the use of glucocorticoids as treatment for mild asthma was shown to improve growth in female fetuses, suggesting that the sex-specific growth impairment is associated with inflammation pathways^11^. It was demonstrated that placentas of asthmatic pregnant women with female (but not male) fetuses had an increased expression of pro-inflammatory cytokines, namely tumor necrosis factor α (TNFα) and interleukin 6 (IL-6), compared to healthy women^12^. Similarly, lipopolysaccharide-stimulated peripheral blood mononuclear cells obtained from women pregnant with females released more IL-6, TNFα and IL-1β than cells from women pregnant with males^12,13^. These findings suggest that the fetus and/or its placenta could influence maternal physiology in a sex-specific manner.

Little is known about the effect of fetal sex on the risk of infection in the mother (the ‘host’), with most studies focusing on whether associations between maternal infection (e.g., malaria) and infant health outcomes (e.g., malaria incidence) differ by sex^14^. In women infected with severe acute respiratory syndrome coronavirus 2 (SARS-CoV-2), maternal SARS-CoV-2-specific antibody titers were lower when the mother carried a male fetus^15^. So far, only one study has focused on maternal malaria risk. Adam et al. studied the relationship between placental malaria and fetal sex in women in eastern Sudan. Placental malaria was detected on placental histology and 88% of infections were past infections. The odds of placental malaria were 2.55 times higher when the fetus was female^16^. To complement the findings of this study, we conducted a meta-analysis of data collected from cohort studies and clinical trials that followed pregnant women in *P. falciparum*-endemic areas ^17^, to determine the association between fetal sex and malaria infection during pregnancy.

## Results

### Characteristics of study population

Of the 13 studies in the original pooled dataset, two studies were excluded. One was unavailable for this meta-analysis, and one did not record maternal malarial infection by LM, PCR or histology^18,19^. Of the remaining 11 studies included, five were observational and six were clinical trials measuring the effect of chemoprevention or insecticide-treated nets^20–31^. Nine studies were conducted in sub-Saharan African countries and two in PNG. Among 12,830 women enrolled in these studies, 12,821 singleton pregnancies with data on fetal sex were included in this analysis.

Approximately two-thirds of the women included were aged 24 years or younger. Gravidity, trimester at antenatal enrolment, and bed net ownership varied across studies. Most of the participants lived in rural areas. In the studies where chemoprevention was used, the most commonly used strategy was intermittent preventive treatment with sulfadoxine-pyrimethamine. Details of each study participants’ characteristics are outlined in Table 1.

**Table 1:** Characteristics of study participants in the 11 studies which were part of the M3 Initiative (n = 12,821) ^17^.

	Benin-Stoppam^20^	Congo-Landis^21^	EMEP-MON ^22,23^	ISTp-Malawi ^48^	Kenya-Ayisi ^25^	Kenya-2 ^26^	Malawi-LAIS ^29^	PNG-IPTp^27^	PNG-Sek ^28^	STOPMIP-Kenya^30^	Burkina Faso ^31^
Sample size	791	164	471	1618	3388	711	1190	1943	293	1228	1026
Maternal age	26.4 [6.1]	27.5 [5.3]	25.3 [6.4]	22.5 [5.1]	21.7 [4.6]	25.8 [6.7]	25 [6.4]	24.5 [5.5]	25.1 [5.6]	23.4 [5.7]	24.4 [6.2)
Gravidity											
1 (Primigravid)	147 (19.9)	43 (26.2)	94 (20.0)	551 (34.1)	1656 (48.9)	127 (17.9)	267 (22.4)	966 (47.5)	115 (39.2)	411 (33.4)	210 (20.5)
2 (Secundigravid)	173 (21.9)	22 (13.4)	77 (16.3)	450 (27.8)	748 (22.1)	118 (16.6)	213 (17.9)	494 (24.3)	54 (18.4)	242 (19.7)	216 (21.3)
3+ (Multigravid)	471 (59.5)	99 (60.4)	300 (63.7)	617 (38.1)	984 (29.0)	466 (65.5)	710 (59.7)	573 (28.2)	124 (42.3)	576 (46.9)	606 (59.1)
Trimester at enrolment*											
1	174 (22.4)	6 (3.7)	69 (14.7)	0 (0)	0 (0)	53 (9.1)	0 (0)	103 (7.9)	2 (0.8)	23 (1.9)	388 (37.9)
2	616 (77.9)	158 (96.3)	260 (55.4)	1601 (99.0)	0 (0)	326 (56.0)	1190 (100)	1749 (90.1)	212 (84.1)	1011 (84.5)	596 (58.2)
3	1 (0.1)	0 (0)	140 (29.9)	17 (1.1)	3388 (100)	203 (34.9)	0 (0)	90 (4.6)	38 (15.1)	194 (15.8)	40 (3.9)
Bed net use											
Yes	254 (32.1)	164 (100)	N/A	331 (20.5)	N/A	348 (48.9)	877 (73.7	1798 (92.5)	240 (83.0)	690 (56.1)	N/A
No	537 (67.9)	0 (0)	N/A	1287 (79.5)	N/A	363 (51.1)	313 (26.3)	145 (7.5)	49 (17.0)	539 (43.9)	N/A
Rural residents	791 (100)	0 (0)	471 (100)	1605 (99.2)	722 (21.3)	711 (100)	1190 (100)	1185 (61)	282 (96.20)	1050 (85.40)	N/A
Chemo-prevention	IPTp-SP	IPTp-SP	IPTp-SP	IPTp-SP, ISTp-DP	–	IPTp-SP	ITp-SP, IPTp-SPAZ	SPCQ, IPTp-SPAZ	SPCQ	IPTp-SP, IPTp-DP, ISTp-DP	IPTp-SP
No of IPTp doses (median)	2	2	N/A	4	N/A	N/A	4		N/A	2	2
Female infant	394 (49.1)	85 (51.8)	249 (52.9)	810 (50.1)	1697 (50.1)	358 (50.4)	584 (49.1)	1078 (55.5)	141 (48.1)	605 (49.3)	514 (50.2)

Data are n (%) or mean [SD].

*ISTp* intermittent screen and treat in pregnancy, *IPTp* intermittent preventive treatment in pregnancy, *DP* dihydroartemisinin-piperaquine, *N/A* not available, *SP* sulphadoxine-pyrimethamine, *SPAZ* SP plus azithromycin, *SPCQ* SP plus chloroquine (single course SP plus CQ at enrolment, followed by weekly CQ).

*Pregnancies were dated by ultrasound or (when ultrasound was not available) symphysis-fundal height. Studies that determined gestational age at first antenatal visit using symphysis-fundal height exclusively include Kenya-Ayisi, Kenya-2 and PNG-Sek.

### Effect of fetal sex on risk of malaria in pregnancy at antenatal enrolment

**Figure 1:**
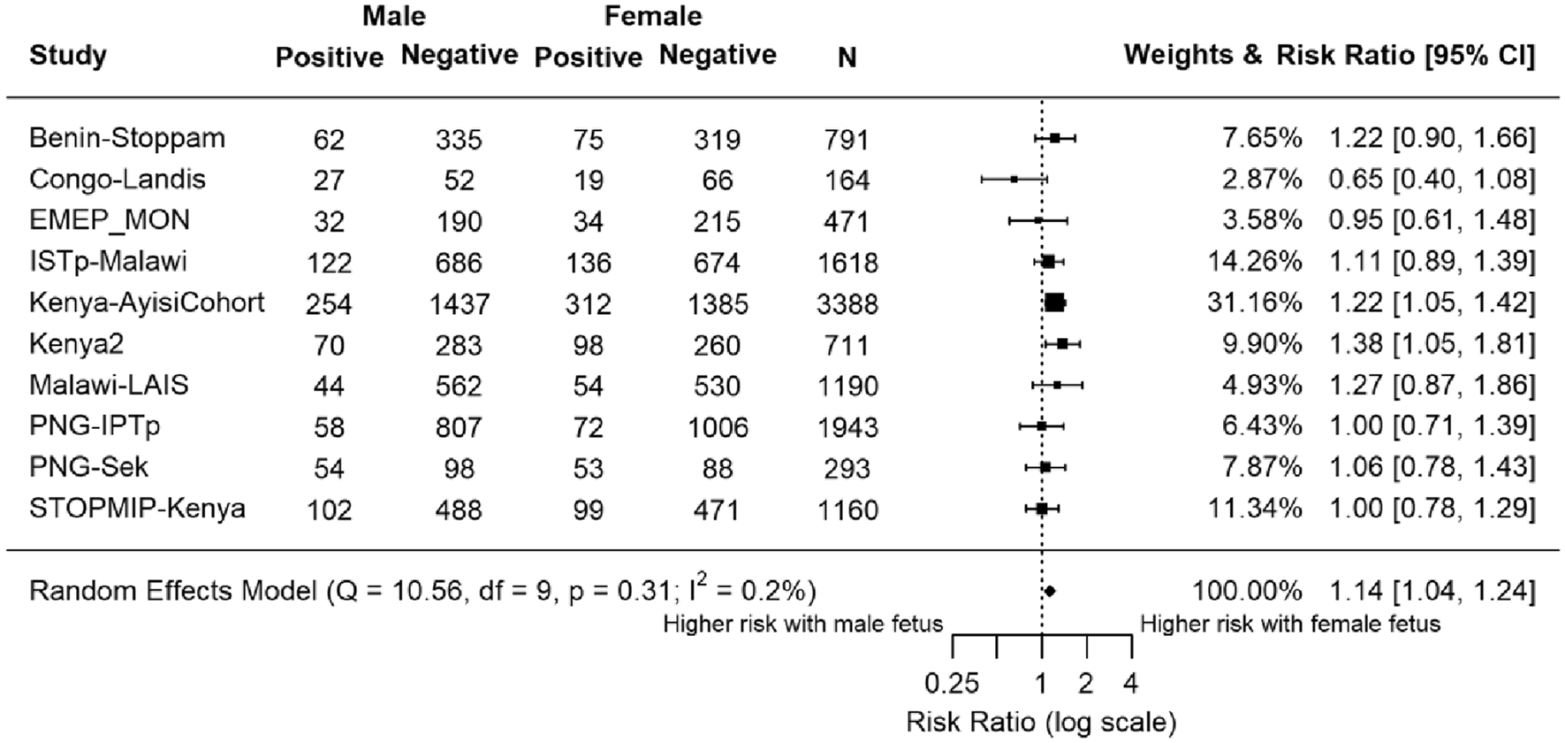
Forest plot of the association between fetal sex and risk of maternal peripheral malaria infection at antenatal enrolment, by light microscopy. Estimates compared the risk of maternal malaria infection by light microscopy at antenatal clinic enrolment in women carrying female fetuses to those carrying male fetuses. Estimates represented by a small box, where the width of the whisker corresponds to the 95% confidence interval (CI). Size of the box is proportional to the weight of the study. Heterogeneity of studies was not statistically significant (P = 0.3, I^2^ = 0.2%). N = 11,729.

Ten studies (n = 11,729) assessed peripheral malaria status at antenatal enrolment using LM (Fig. 1). Overall, the summary estimate RR was 1.14, suggesting a higher risk of malaria with female fetuses (95% confidence interval [CI] 1.04, 1.24; P = 0.0032). Most studies showed an increased prevalence of peripheral malaria infection at antenatal enrolment in mothers carrying female fetuses (Supplementary Fig. 1). Seven studies found an increased risk of malaria infection associated with carrying a female fetus, although differences were only statistically significant at P < 0.05 for two individual studies, both from Kenya (Fig. 1).

Four studies (n = 4976) assessed peripheral malaria infection status at enrolment using PCR (Supplementary Fig. 2). The overall change in risk was minimal across all four studies. The summary estimate for this marker was 1.01 (95% CI 0.94, 1.04; P = 0.83).

### Effect of fetal sex on risk of peripheral malaria infection at delivery

**Figure 2:**
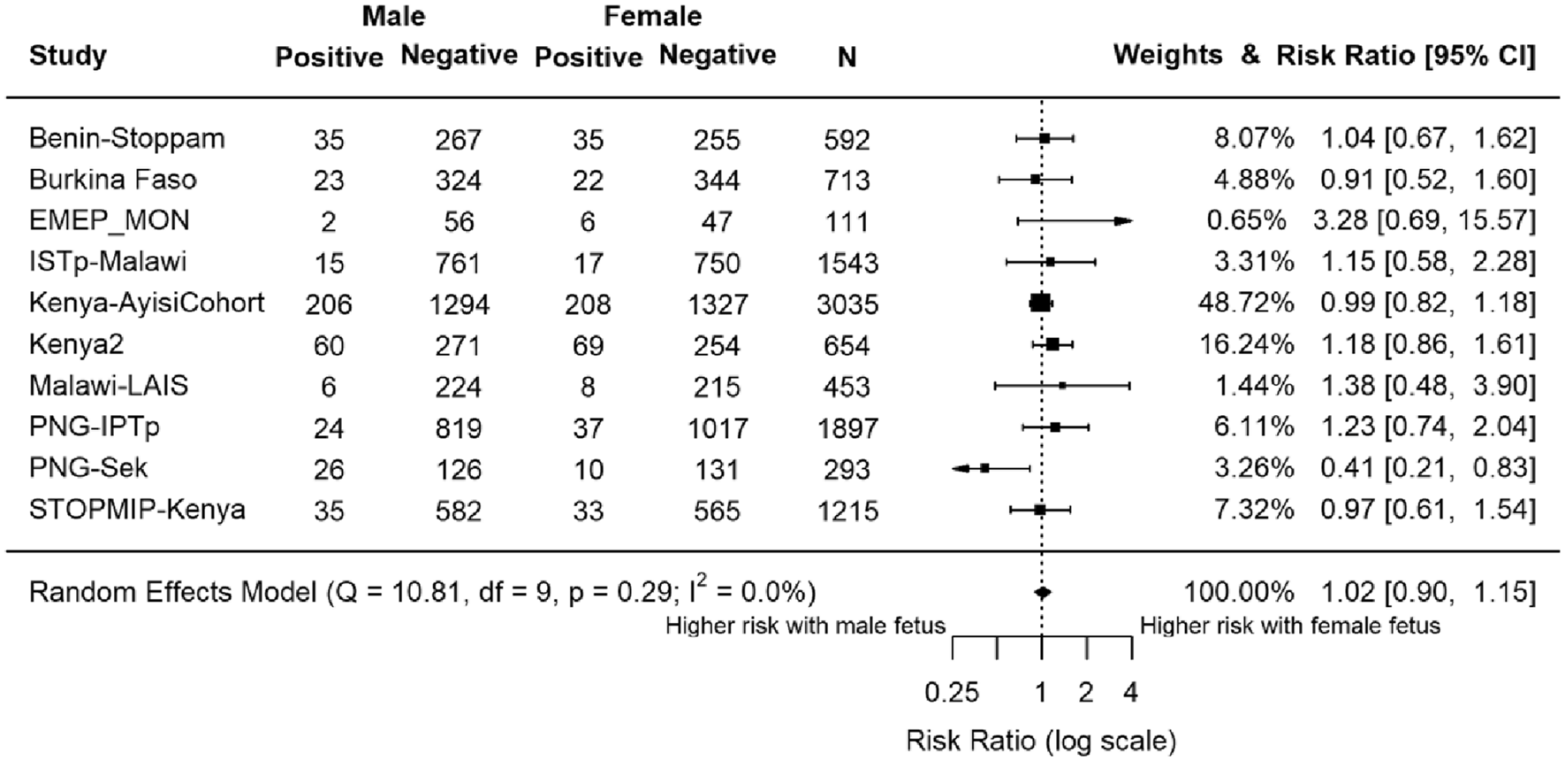
Forest plot of the association between fetal sex and risk of maternal peripheral malaria infection at delivery, by light microscopy. Estimates compared the risk of maternal malaria in those carrying female fetuses to those carrying male fetuses. Estimates represented by a small box, where the width of the whisker corresponds to the 95% confidence interval (CI). Size of the box is proportional to the weight of the study. Heterogeneity of studies not statistically significant (P = 0.29, I^2^ = 0.0%). *N* = 10,506.

Peripheral malaria infection at delivery was diagnosed by LM in 10 studies (n = 10,506) (Fig. 2). Overall, there was no difference in maternal malaria risk by LM at delivery by fetal sex (RR 1.02, 95% CI 0.90, 1.15; P = 0.78). In six studies, malaria risk was higher with a female fetus and in four it was higher with a male fetus (Fig. 2). The PNG-Sek study showed that women carrying a female fetus had lower risk of malaria at delivery (RR 0.41, 95% CI 0.21, 0.83, P = 0.009).

Four studies (n = 2739) assessed peripheral malaria infection at delivery by PCR. Two found a heightened risk with a female fetus, while the other two did not, but none of these differences were statistically significant (Supplementary Fig. 3). The summary estimate for this marker was 1.03 (95% CI 0.80, 1.33; P = 0.80).

### Effect of fetal sex on risk of placental malaria

**Figure 3:**
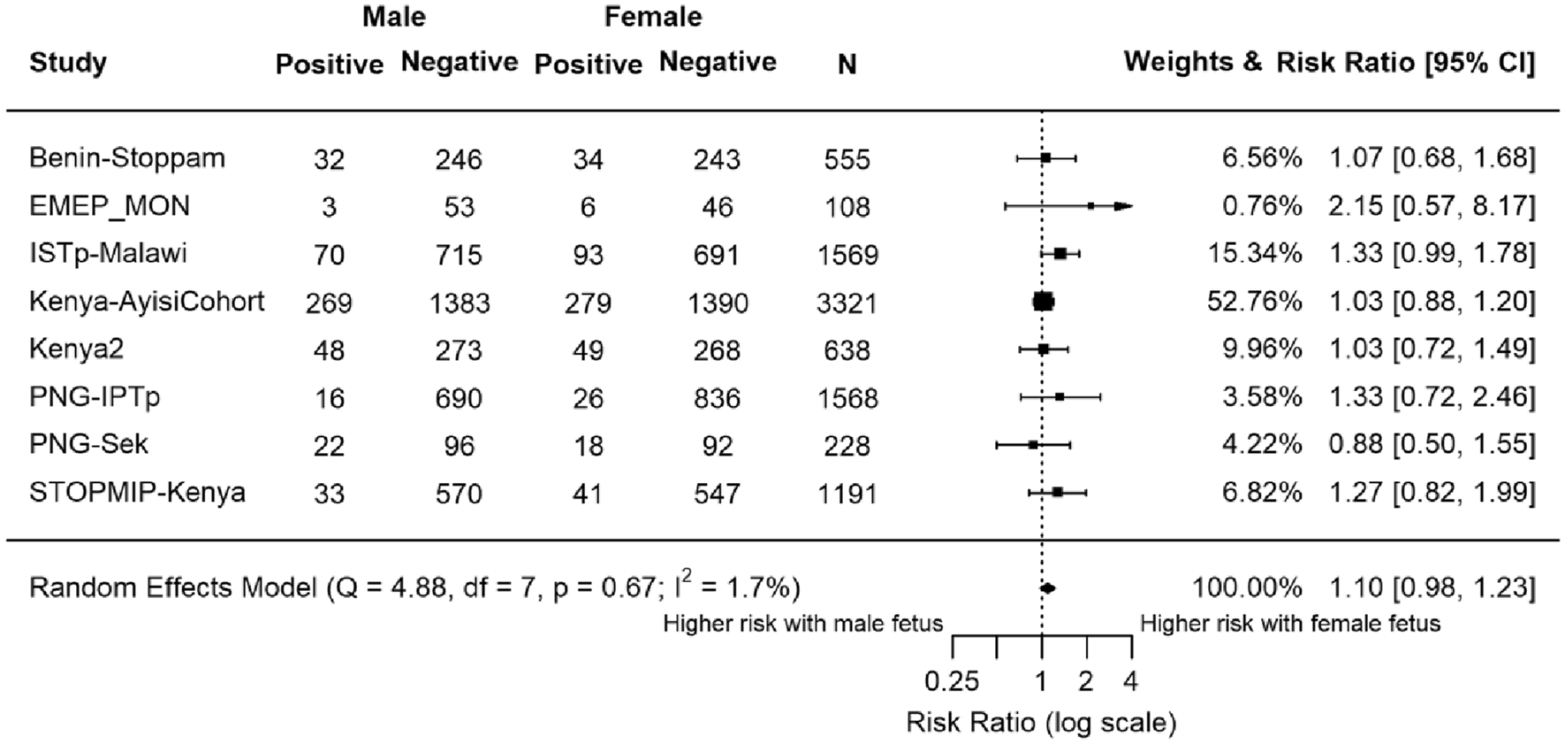
Forest plot of the association between fetal sex and risk of placental malaria tested at delivery by light microscopy. Estimates compared the risk of placental malaria infection in women carrying female fetuses to those carrying male fetuses. Estimates represented by a small box, where the width of the whisker corresponds to the 95% CI. Size of the box is proportional to the weight of the study. Heterogeneity of studies not statistically significant (P = 0.67, I^2^ = 1.7%). *N* = 9178.

Placental malaria was assessed by LM in eight studies (n = 9178; Fig. 3). Estimates of seven studies indicated heightened risk of malaria in women carrying female fetuses. Overall, the summary estimate was 1.10 (95% CI 0.98, 1.23; P = 0.12).

Four studies (n = 4113) assessed for placental malaria infection at delivery using PCR (Supplementary Fig. 4). The summary estimate of this marker was 1.09 (95% CI 0.95, 1.27; P = 0.23).

**Figure 4:**
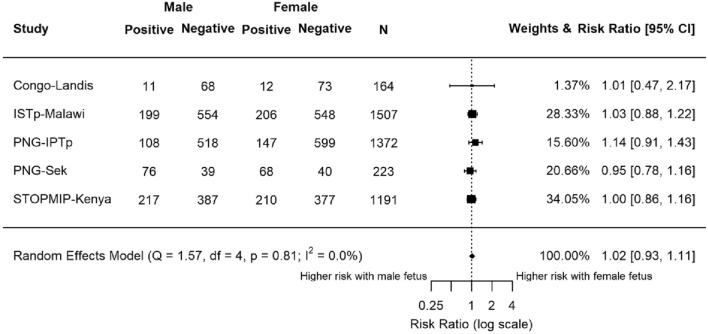
Forest plot of the association between fetal sex and risk of placental malaria tested at delivery by histology. Active and past infections were both included. Estimates compared the risk of placental malaria in women carrying female fetuses to those carrying male fetuses. Estimates represented by a small box, where the width of the whisker corresponds to the 95% CI. Size of the box is proportional to the weight of the study. Heterogeneity of studies not statistically significant (P = 0.81, I^2^ = 0.0%). *N* = 4457.

Placental malaria at delivery was assessed by histology in five studies (n = 4457; Fig. 4). We considered both active (n = 468) and past infections (n = 786) as indicative of placental infection. The summary estimate for this marker was 1.02 (95% CI 0.93, 1.11; P = 0.68). Neither the meta-analysis nor any individual study detected a statistically significant association between fetal sex and placental malaria detected by histology.

## Discussion

This study aimed to determine the impact of fetal sex on the risk of malaria in pregnancy. We found that women carrying female fetuses were at higher risk of peripheral malaria infection detected by microscopy at antenatal enrolment (RR 1.14). This was the only one out of seven malaria markers that showed a statistically significant result in meta-analysis, but the directionality of effect was similar when placental infection detected by LM at delivery was considered (RR 1.10). At the individual study level, one small cohort study suggested an association with male sex and increased risk of peripheral infection at delivery, but not other malaria markers^28^.

Light microscopy at antenatal enrolment was the most frequently measured marker of malaria infection, i.e., many of the included studies used LM to diagnose malaria infection, while fewer used PCR and histology. The large sample size for LM at enrolment contributes to a higher statistical power, reducing the probability of incorrectly accepting the null hypothesis^32^. Of the ten individual studies where malaria diagnosed by LM at antenatal enrolment was available, fetal sex was statistically significantly associated with malaria infection in two studies, both conducted in western Kenya in the 1990s when malaria transmission was very high, and there was a trend towards increased risk in another three studies (Fig. 1). An association between fetal sex and maternal malaria infection risk may be most discernible at antenatal enrolment, i.e., prior to the provision of various malaria interventions including antimalarial drugs and insecticide-treated nets that were evaluated in participating studies, and that are likely to alter malaria risk.

To date, only one study has explored the interaction between fetal sex and malaria risk in pregnancy. The study followed 339 women living in eastern Sudan, where intermittent preventive treatment in pregnancy (IPTp) is not used, and assessed placental malaria by histology. Risk of placental malaria was reported to be 2.55 times higher in women carrying female fetuses^16^. We were unable to replicate an association between female sex and placental infection on histology, perhaps because IPTp or intermittent screening and treatment was used in 10 of 11 studies included in this meta-analysis. However, our findings add to the evidence that carrying a female fetus may be associated with a higher risk of malaria parasitaemia in pregnancy. The disparate finding of increased risk of peripheral infection by light microscopy in male babies in a small cohort study from PNG may relate to sample size, and possibly loss-to-follow-up and documentation bias. There may also be sex-specific differences in risk of malaria in infancy. Severe past placental malaria (presence of pigment in > 20% of high power fields by microscopy of placental histology) was associated with an increased incidence of malaria in infancy in male but not female babies^14^, and IPTp with dihydroartemisinin-piperaquine was found to reduce incidence of malaria in male but not female infants^33,34^. These studies suggest that fetal sex might play a role in modulating malaria risk in infancy that is associated with placental malaria.

The mechanisms underpinning the potential association between female fetuses and malaria infection risk in the mother have yet to be elucidated. The placenta acts as an endocrine organ, secreting hormones and cytokines that might affect the physiology of both the mother and the fetus^35,36^. Several studies found that X chromosome inactivation results in sex-dependent differential gene expression, in both autosomal and sex-linked genes^35,37^. This includes upregulation of certain immunological mediators, including TNFα, in female placentas^38^. Previously, high levels of TNFα have been associated with higher parasite density and slower clearance^39,40^. Perhaps this upregulation of TNFα receptors led to an increased sensitivity to TNFα, resulting in slower clearance of parasites and more detectable infections in mothers carrying female fetuses. Asthma studies have shown that female placentas tend to upregulate cortisol levels in response to changes in maternal cortisol levels, which eventually results in increased expression of TNFα^12,41^. Possible mechanisms include sex-differential function of placental glucocorticoid receptor isoforms and reductions in placental 11β-hydroxysteroid dehydrogenase type 2 expression^41,42^. Further studies are required to confirm if a similar mechanism is observed with malaria infection. Upregulation of receptors used by parasites to sequester in the placenta should also be investigated as a possible mechanism in future studies.

The present study, and the preceding study by Adam et al., considered women who had a live born baby only. Male fetuses are at increased risk of stillbirth, and malaria is associated with stillbirth and miscarriage^43–45^. It is plausible that malaria in early gestation increases the risk of male fetal loss, which may manifest in an increased prevalence of malaria in pregnant women carrying female fetuses.

This study had strengths and limitations. Compared to the study by Adam et al.^16^, the dataset we used was of substantially larger sample size, including 4457 women for whom placental histology was available. The study population was drawn from a wider geographical area, including Papua New Guinea and multiple sub-Saharan African countries. This allows for more generalisation of the findings to *P. falciparum*-endemic regions. Furthermore, infection was assessed using LM, PCR and histology. The addition of PCR as a diagnostic tool enabled the detection of sub-microscopic infections. However, only four out of 11 studies tested for malaria infection using PCR, and the meta-analysis lacked power to conclusively evaluate the association between fetal sex and submicroscopic infection. Furthermore, it might be that fetal sex contributes to regulation of parasite density, rather than incidence of infection, and this could manifest in differences in infections detectable by microscopy, but not infections detected by PCR. The M3 initiative did not collate information on parasite densities. Lastly, differences in malaria risk, or the apparent lack of an association between fetal sex and malaria risk at birth, may have been the result of interventions started at antenatal enrolment such intermittent preventive treatment or provision of insecticide treated nets. In order to yield more conclusive results, assessing associations between fetal sex and malaria infection may be strengthened by including more studies that screened for infection, e.g., at enrolment using both LM and PCR, and that include pregnancies that resulted in stillbirth or miscarriage. Moreover, as the study population was diverse, other variables such as the maternal age and gravidity could have been confounders.

In conclusion, carrying a female fetus may be associated with a higher risk of malaria in pregnancy, as previously reported, but the magnitude of any such risk is modest. Women with a female fetus had a higher risk of peripheral malaria infection at antenatal enrolment when determined by LM. This time point included the largest number of observations, and preceded the initiation of interventions such as IPTp or insecticide-treated bed nets that could have potentially masked sex-differential effects. Individual participant data meta-analysis of more recent interventional and cohort studies, adjusting for potential confounders and using ultrasound dating and including fetal losses, could confirm our findings, while efforts should be made to identify the underlying biological mechanisms that could underpin fetal sex-based differences in the risk of malaria in pregnancy.

## Methods

### Study population

The study population, summarised in detail elsewhere^17^, was derived from a dataset gathered by the Maternal Malaria and Malnutrition (M3) initiative that originally included data pooled from 13 studies following 13,898 pregnant women in *P. falciparum*-endemic areas, including Papua New Guinea (PNG) and several countries in sub-Saharan Africa (see cohort profile)^17^. Studies included were either prospective cohort studies or randomised controlled trials conducted between 1996 and 2015. Variables such as age, gravidity, and body mass index of the participants were recorded. Participants were also assessed for malaria infection at antenatal clinic enrolment and at delivery, using various diagnostic tools including light microscopy (LM) of blood films and polymerase chain reaction (PCR). Most studies used LM to detect parasitaemia, while some also used PCR to detect sub-microscopic infections. At delivery, some studies assessed the presence of placental malaria using LM, PCR or placental histology^17^, and each was analysed individually.

Where available, placental histology results were classified into three categories. Active infection was characterised by presence of parasites, regardless of presence of pigment in fibrin. Past infection was characterised by absence of parasites and presence of fibrin pigment. No infection was characterised by absence of both parasites and fibrin pigment^46^.

### Data analysis

Univariable meta-analysis was performed using RStudio version 4.0.2. The dataset was reshaped for compatibility with the metafor package in R^47^. Women without data for fetal sex were excluded, as were women who had not been screened for malaria infection by LM, PCR or histology.

The exposure of interest was fetal sex, defined as male or female, and the outcome measure was malaria infection, assessed using differing techniques at enrolment or delivery. The association between fetal sex and maternal malaria infection was investigated for peripheral malaria infection detected by LM and PCR at enrolment and delivery, and for placental malaria detected by LM, PCR and placental histology. We calculated risk ratios and sampling variances using log-binomial regression analyses (escalc function) and fitted them to a random-effects model to calculate the summary estimate and heterogeneity (rma function). Forest plots were generated using the forest function. Heterogeneity was assessed using the P-value of the Chi-square statistic and I^2^. A P-value of less than 0.1 and I^2^ value of more than 40% were considered significant. Rows with missing values for respective malaria infection indicators were omitted. In meta-analyses by time-point and diagnostic modality, studies that had less than 100 women with data for a respective malaria indicator of interest were also excluded, as were studies that had no women with malaria in at least one of the groups (male or female fetus). To account for multiple comparisons (n = 7) a Bonferroni-corrected P-value of < 0.007 was used to denote statistical significance.

All studies received approval by their local ethics board and obtained informed consent from all participants. Details of relevant procedures are highlighted in the published cohort profile ^17^.

## Supplementary Information

Supplementary Figures.

## Data Availability

Data are available from the WWARN data repository (http://www.wwarn.org/working-together/sharing-data/accessing-data) for researchers who meet the criteria for access to confidential data.
