# Supplementary material for: Fetal sex and risk of pregnancy-associated malaria in Plasmodium falciparum-endemic regions: a meta-analysis
Source: Sci Rep. 2023 Jun 26;13:10310. doi: 10.1038/s41598-023-37431-3 (PMC10293221; doi:10.1038/s41598-023-37431-3)
Supplement: Supplementary file 1 — Supplementary Figures. [file 41598_2023_37431_MOESM1_ESM.docx]

**Supplemental Figures**

**
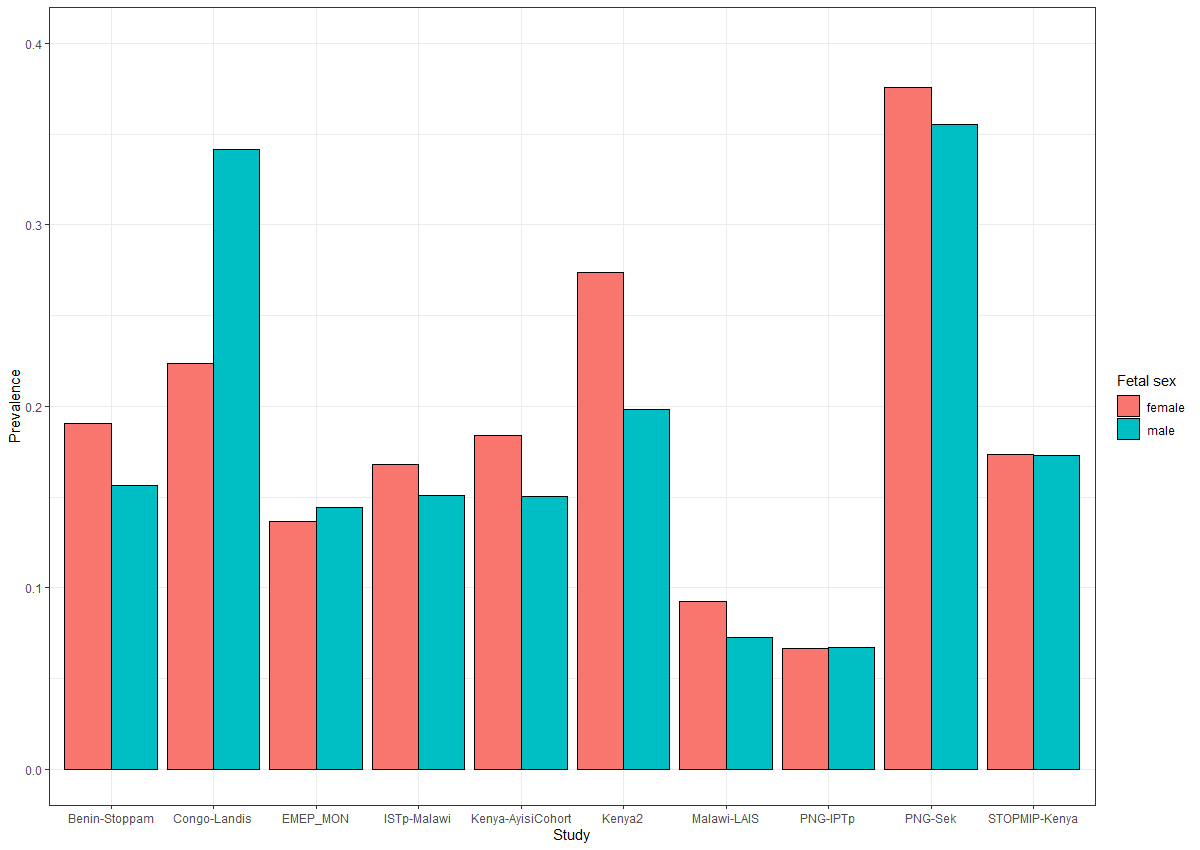
**

**Supplemental Figure 1. Prevalence of malaria infection at antenatal enrolment as detected by light microscopy, by fetal sex.** Pink bars represent women carrying a female fetus. Blue bars represent women carrying a male fetus. Seven studies showed higher prevalence of peripheral malaria infection in mothers carrying female fetuses, two studies showed higher prevalence in mothers carrying male fetuses, and two studies showed equal prevalence regardless of fetal sex.

**
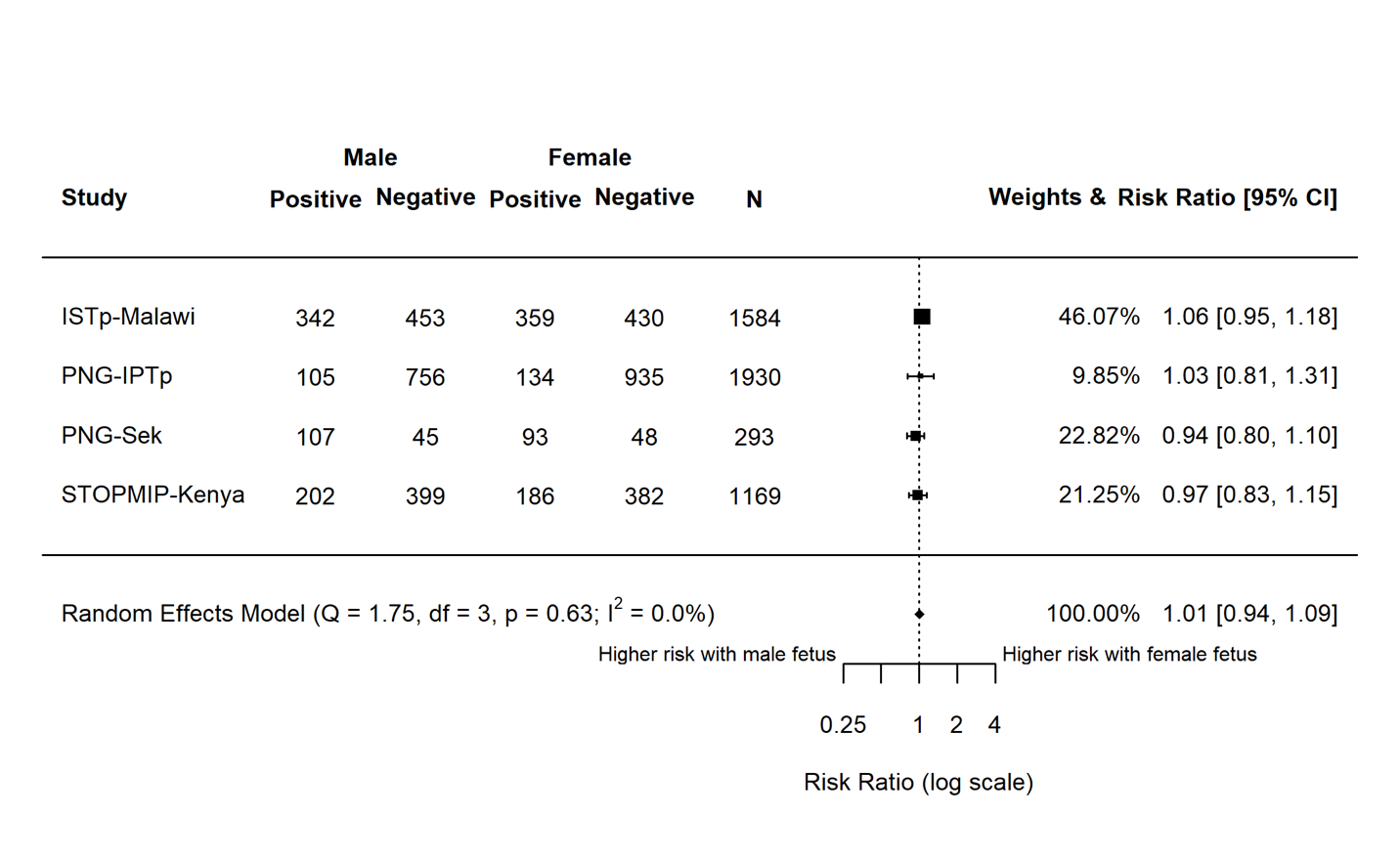
Supplemental Figure 2.** **Forest plot of the association between fetal sex and risk of peripheral malaria infection at antenatal enrolment, by polymerase chain reaction**. Estimates compared the risk of maternal malaria infection in women carrying female fetuses to those carrying male fetuses. Estimates represented by a small box, where the width of the whisker corresponds to the 95% CI. Size of the box is proportional to the weight of the study. Heterogeneity of studies not statistically significant (P=0.63, I^2^ = 0.0%). N=4,976.

**
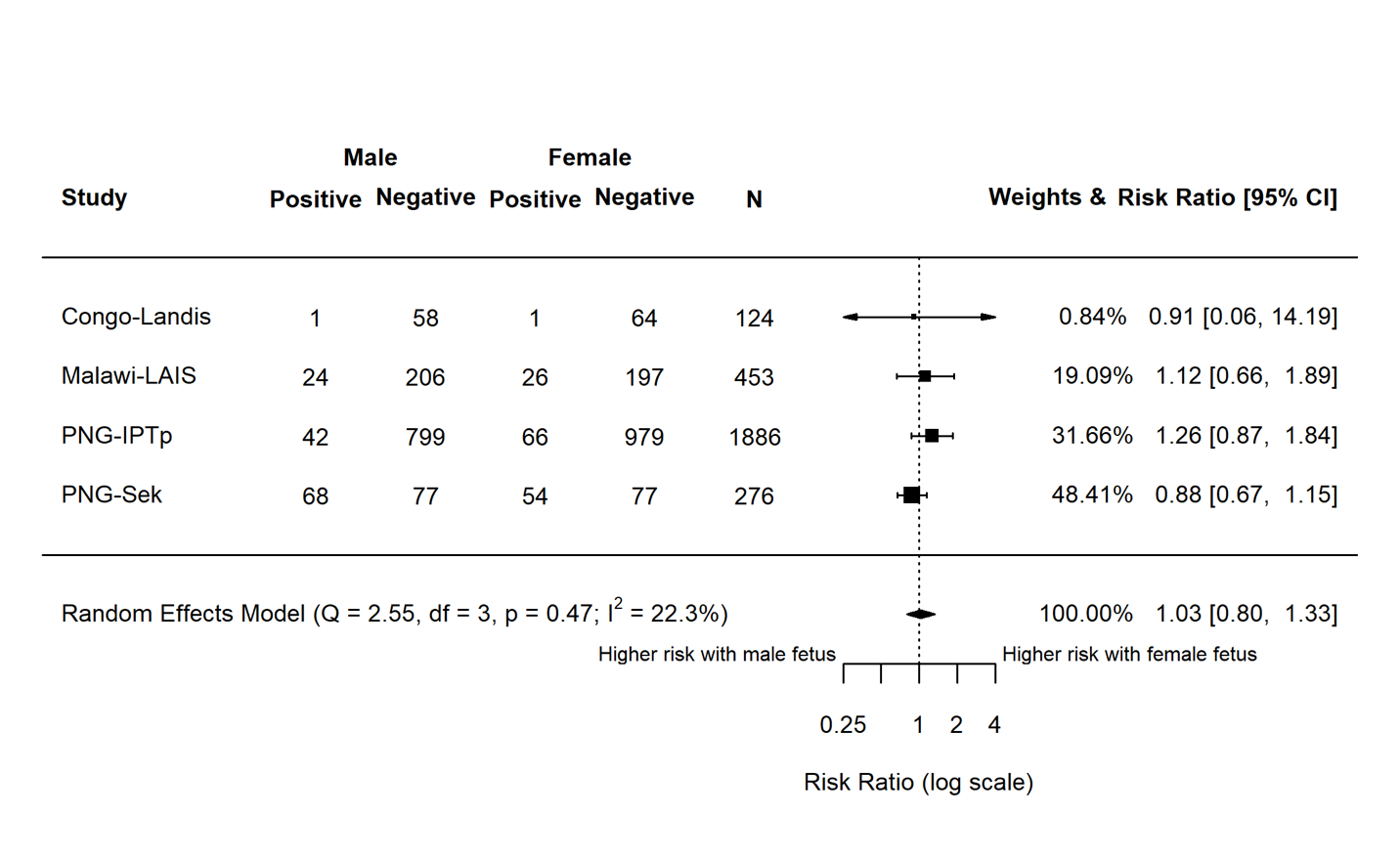
Supplemental Figure 3.** **Forest plot of the association between fetal sex and risk of peripheral malaria tested at delivery by PCR**. Estimates compared the risk of maternal malaria infection in women carrying female fetuses to those carrying male fetuses. Estimates represented by a small box, where the width of the whisker corresponds to the 95% CI. Size of the box is proportional to the weight of the study. Heterogeneity of studies not statistically significant (P=0.47, I^2^ = 22.3%). N=2,739.

**
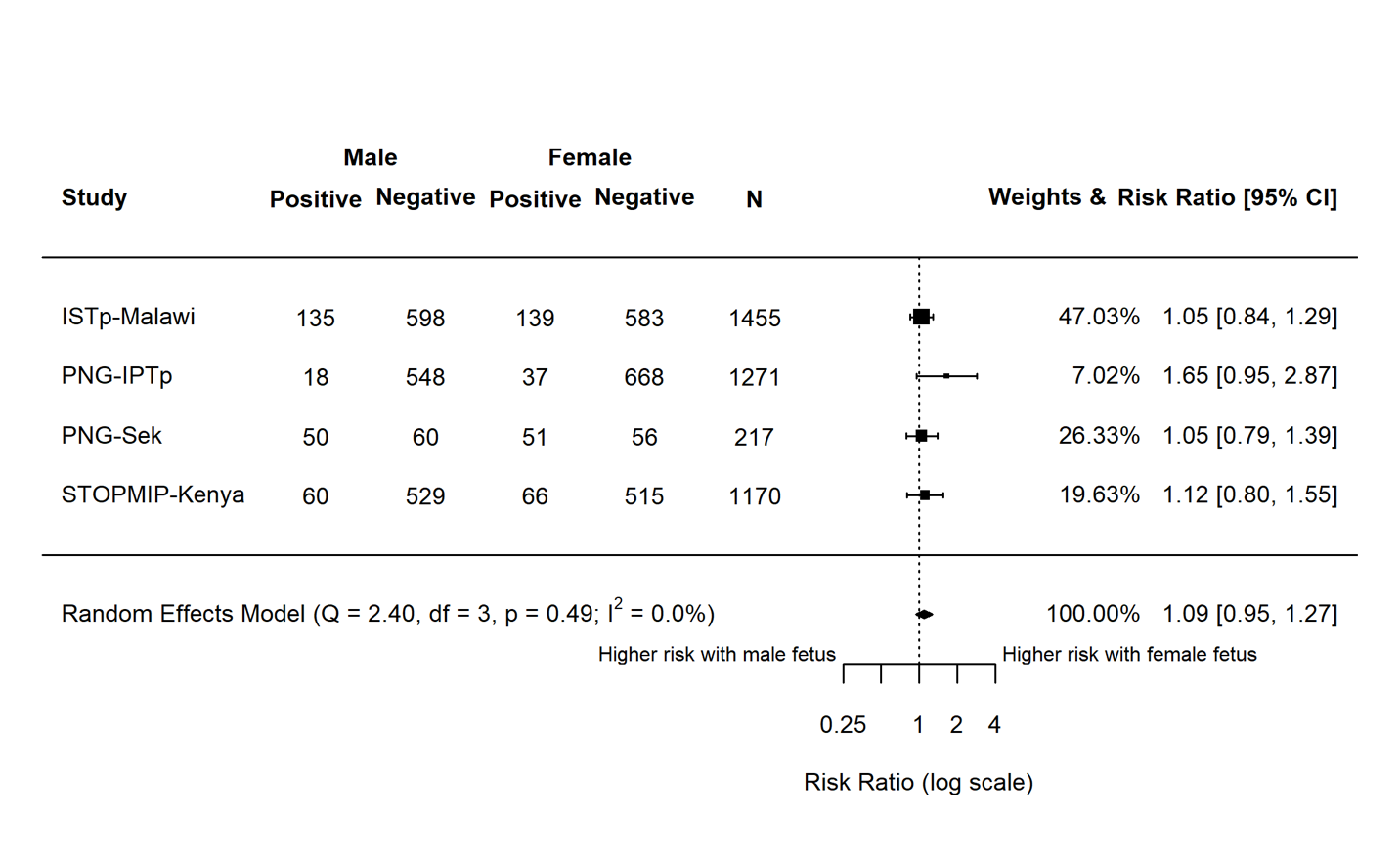
Supplemental Figure 4.** **Forest plot of the association between fetal sex and risk of placental malaria tested at delivery by PCR**. Estimates compared the risk of placental malaria infection in women carrying female fetuses to those carrying male fetuses. Estimates represented by a small box, where the width of the whisker corresponds to the 95% CI. Size of the box is proportional to the weight of the study. Heterogeneity of studies not statistically significant (P=0.49, I^2^ = 0.0%). *N*=4,113.
